# Detrimental effect of post *Status Epilepticus* treatment with ROCK inhibitor Y-27632 in a pilocarpine model of temporal lobe epilepsy

**DOI:** 10.3389/fncel.2015.00413

**Published:** 2015-10-23

**Authors:** Nazim Kourdougli, Saara Varpula, Genevieve Chazal, Claudio Rivera

**Affiliations:** ^1^INSERM Unité 901, INMEDMarseille, France; ^2^Aix-Marseille Université, UMR S901Marseille, France; ^3^Neuroscience Center, University of HelsinkiHelsinki, Finland

**Keywords:** temporal lobe epilepsy, ROCK inhibitor, epileptogenesis, astrogliosis, KCC2, mossy fiber sprouting

## Abstract

Temporal lobe epilepsy (TLE) is the most common type of epilepsy in adults where 20–30% of the patients are refractory to currently available anti-epileptic drugs. The RhoA/Rho-kinase signaling pathway activation has been involved in inflammatory responses, neurite outgrowth and neuronal death under pathological conditions such as epileptic insults. Acute preventive administration of ROCK inhibitor has been reported to have beneficial outcomes in *Status Epilepticus* (SE) epilepsy. In the present study, we evaluate the effect of chronic post SE treatment with the ROCK inhibitor Y-27632 in a rat pilocarpine model of TLE. We used chronic i.p. injections of Y-27632 for 5 days in 6 week old control rats or rats subjected to pilocarpine treatment as a model of TLE. Surprisingly, our findings demonstrate that a systemic administration of Y-27632 in pilocarpine-treated rats increases neuronal death in the CA3 region and ectopic recurrent mossy fiber sprouting (rMFS) in the dentate gyrus of the hippocampal formation. Interestingly, we found that chronic treatment with Y-27632 exacerbates the down-regulation and pathological distribution of the K^+^-Cl^−^ cotransporter KCC2, thus providing a putative mechanism for post SE induced neuronal death. The involvement of astrogliosis in this mechanism appears to be intricate as ROCK inhibition reduces reactive astrogliosis in pilocarpine rats. Conversely, in control rats, chronic Y-27632 treatment increases astrogliosis. Together, our findings suggest that Y-27632 has a detrimental effect when chronically used post SE in a rat pilocarpine model of TLE.

## Introduction

Temporal lobe epilepsy (TLE) is the most common type of epilepsy in adults where 20–30% of the patients are refractory to currently available anti-epileptic drugs (Leonardi and Ustun, 2002; Engel et al., 2012). After an initial insult, both in humans and animal models, TLE is characterized by a clinically quiescent period followed by the occurrence of spontaneous and recurrent seizures (Cavalheiro et al., 1991; Dudek and Sutula, 2007; Curia et al., 2008). The limbic system and, in particular, the hippocampus is one of the major seizure generation loci (McNamara, 1994; Toyoda et al., 2013). During epileptogenesis, the hippocampus displays substantial morphological and functional reorganizations such as excitotoxicity-mediated cell death (Turski et al., 1983; Curia et al., 2008; Bernhardt et al., 2013), reactive gliosis (do Nascimento et al., 2012; Rossi et al., 2013; Hadera et al., 2015) and subsequent ectopic recurrent mossy fiber sprouting (rMFS; Tauck and Nadler, 1985; Represa et al., 1989; Sutula et al., 1989; Mathern et al., 1999; Gabriel et al., 2004; de Lanerolle et al., 2012; Peret et al., 2014) as well as GABAergic network rewiring (Sloviter, 1987; Cossart et al., 2005). The signaling pathways and underlying cellular and molecular mechanisms involved in this reactive plasticity are of particular importance since they create a powerful hyperexcitable cerebral focus that drives recurrent disabling seizures (McNamara, 1994; Morimoto et al., 2004; Sloviter, 2008; Jefferys, 2010; Paz and Huguenard, 2015).

The RhoA/Rho-kinase (Rho-ROCK) signaling pathway is highly activated under pathological conditions such as spinal cord injury, ischemia and post-traumatic epilepsy (Fournier et al., 2003; Dubreuil et al., 2006; Ding et al., 2010). Recently, the Rho-ROCK pathways have been involved in numerous neuronal functions such as microglial and astroglial inflammatory responses, neurite outgrowth and neuronal survival within the central nervous system (for review, see Kubo et al., 2008; Fujita and Yamashita, 2014; Hensel et al., 2015). For instance, chronic treatment using ROCK inhibitors such as fasudil or Y-27632 improved recovery after spinal cord injury (Fournier et al., 2003; Boomkamp et al., 2012; Joshi et al., 2015). Also, acute treatment with fasudil or Y-27632 promotes neuronal survival and restrains inflammatory responses in models of ischemia and epilepsy when used prior to insult both *in vitro* and *in vivo* (Yamashita et al., 2007; İnan and Büyükafşar, 2008; Ding et al., 2010; Gisselsson et al., 2010).

Under pathophysiological conditions, GABA_A_-mediated responses become depolarizing (for review, see Medina et al., 2014). This is believed to participate in epileptogenic processes. This includes the deregulation of functional expression of the neuronal specific K^+^-Cl^−^ co-transporter (KCC2) as reported in resected tissues from human TLE patients (Palma et al., 2006; Huberfeld et al., 2007; Muñoz et al., 2007) and animal models of TLE (Pathak et al., 2007; Bragin et al., 2009; Barmashenko et al., 2011) as well as following stroke or traumatic brain injury (Bonislawski et al., 2007; Jaenisch et al., 2010). In addition, recent findings show that human mutations altering KCC2 expression levels have been linked to epilepsy of infancy or iodiopathic epilepsy (Kahle et al., 2014; Puskarjov et al., 2014; Stödberg et al., 2015). Both GABA_A_-mediated depolarization and KCC2 downregulation could participate in post-traumatic processes leading to neuronal death (Shulga et al., 2008, 2012; Pellegrino et al., 2011; Medina et al., 2014; Winkelmann et al., 2015). We have also previously shown that depolarizing GABAergic transmission triggers the activation of the Rho-ROCK signaling pathway, in an acute injury model (Shulga et al., 2012). Considering the involvement of the Rho-ROCK pathway in the pathogenesis of several insults, ROCK seems to be a promising therapeutic target for TLE. However, the effect of ROCK inhibitor treatment during the early time window following *Status Epilepticus (SE)* has not been investigated. In the present study, we tested the effect of chronic treatment using the ROCK inhibitor Y-27632, a relatively specific inhibitor at low concentrations (Davies et al., 2000), during the early epileptogenesis period following SE in a rat pilocarpine model of TLE. We found that chronic treatment using the Y-27632 for 5 days post SE exacerbates the neuronal damage and increases early ectopic rMFS within the hippocampus during early epileptogenesis. Moreover, we show that this was accompanied by a downregulation of KCC2, a major regulator of chloride homeostasis. In conclusion, our findings demonstrate that Y-27632 exacerbates typical features of epileptogenesis which suggest that the ROCK inhibition may not be suitable as an anti-epileptic prophylactic drug in a rodent model of TLE.

## Materials and Methods

### Animals

All experiments were approved by the Institut National de la Santé et de la Recherche Médicale (INSERM), by the local ethic committee (project number 66-15112012) and the European community council directive (2010/63/UE). Adult male Wistar rats (Charles River and Janvier Laboratories) had *ad libitum* access to food and water and were single housed at 22–24°C under a 12 h light/dark cycle.

### Rat Model of Temporal Lobe Epilepsy

Six week old male rats were treated with pilocarpine hydrochloride (360 mg/kg, i.p.; Sigma-Aldrich) 30 min after scopolamine methyl nitrate (0.5 mg/kg, i.p.; Sigma-Aldrich). This pilocarpine dose was chosen because it elicited the most severe seizures with limited mortality, as previously described by other investigators (Esclapez et al., 1999; Artinian et al., 2011). Animals were continuously observed to identify the onset of SE. Pilocarpine injections resulted in continuous, repetitive behavioral seizures (defined as SE). Behavioral rating of seizures used the Racine scale (Racine, 1972). Stage 1 was characterized by behavioral arrest; stage 2 by head nodding, gnawing, and mild tremors; stage 3 by unilateral forelimb clonus; stage 4 by bilateral forelimb clonus and stage 5 by severe seizures with prolonged loss of postural control or prolonged tonus. The onset of SE was defined as the time when animals experienced continuous stage 5 seizures. Rats that did not fulfill these criteria for SE were not included in analyses (Table 1). After 3 h of SE, convulsions were suppressed with diazepam (8 mg/kg, i.p.; Roche) and saline solution (0.9% NaCl; Sigma-Aldrich) was administrated subcutaneously for re-hydratation of the animals. The injection of diazepam was repeated as needed to help terminate behavioral seizures up to 12 h after the end of SE. In the present study, only male rats were subjected to pilocarpine injections, since female rats are resistant to pilocarpine (Persinger et al., 1988; Mejías-Aponte et al., 2002; Scharfman and MacLusky, 2014). The variation of sex hormones is a source of variability as testoterone and estradiol modulate seizure activity in both animal models and human patients (Edwards et al., 1999; Smith et al., 1999, 2002; Valente et al., 2002; Galanopoulou et al., 2003; Scharfman et al., 2005; Scharfman and MacLusky, 2006; Giorgi et al., 2014; D’Amour et al., 2015) as well as behavioral cognitive functions in mice model of TLE (Oliveira et al., 2015). Moreover, few studies have reported that KCC2 expression is sexually dimorphic and controlled by estrogen levels (Galanopoulou and Moshé, 2003; Nakamura et al., 2004; Perrot-Sinal et al., 2007; Galanopoulou, 2008; Giorgi et al., 2014) and that ovarian cycle induces changes in GABA_A_-receptor subunits and subsequent inhibition (Stell et al., 2003; Maguire et al., 2005; Maguire and Mody, 2007). Thus, these phenomenon could alter the response to drug affecting the GABAergic system under epileptic conditions (Scharfman and MacLusky, 2014).

**Table 1:** **Details of animals for pilocarpine induction**.

	Pilo + Vehicle	Pilo + ROCK-I	*P* value
Number of animals	16	24	–
Age (days)	40.42 ± 0.68	39.45 ± 0.95	0.375
Weight (grams)	192.42 ± 6.91	196.6 3 ± 3.53	0.78
Global success rate*	67%	–

All values are mean ± SEM; Mann-Whitney test was used for significance. *To avoid variability each Pilo + Veh and Pilo + ROCK-I pair was taken from the same induction session. The number of animals stated here represents 67% of the animals that successfully went into SE. The original number of rats subjected to pilocarpine was *n* = 47 animals.

### ROCK Inhibitor Y-27632 Treatment

Y-27632 (Y0503, Sigma-Aldrich) was dissolved in 0.9% NaCl and injected intraperitoneally two times per day at a dose of 10 mg/kg in a volume of 0.1 ml per 100 g of body weight. Pilocarpine animals injected with Y-27632 were treated from the 1st day to the 5th day post SE. Pilocarpine-treated vehicle animals received a volume of saline solution (0.1 ml per 100 g of body weight). The saline or ROCK inhibitor Y-27632 treatment was administrated from 8–24 h after the last injection of diazepam (8–24 h after termination of behavioral seizures). Age, weight and strain matching control animals were injected intraperitoneally, two times per day, during 5 days with saline (0.9% NaCl) or Y-27632 at a dose of 10 mg/kg in a volume of 0.1 ml per 100 g of body weight, and received the same total number of injection than pilocarpine-treated rats.

### Immunohistochemstry

Rats were anesthetized with isoflurane prior to the i.p. injection of 40 mg/kg of pentobarbital and transcardiacally perfused with cold phosphate buffer saline (PBS, 1 M) prior to cold 4% paraformaldehyde in PBS. Brains were post-fixed overnight in 4% paraformaldehyde in PBS at 4°C and then coronally cut with a Leica VT1200S Vibratome (60 μm thick-sections). Sections were then permeabilized and blocked in PBS with 0.3% Triton X-100 and 5% normal goat serum (NGS) for 1 h at room temperature. Incubation with primary antibodies diluted in PBS with 5% NGS and 0.1% Triton X-100 was carried out at 4°C overnight using rabbit-KCC2 (home product, Ludwig et al., 2003; 1/4000), mouse-SPO (Synaptoporin, Synaptic Systems, 1/800), mouse-NeuN (Millipore, 1/1000) and rabbit-GFAP (Sigma, 1/1000). After rinsing three times in PBS, slices were incubated with the corresponding Alexa Fluor-conjugated secondary antibodies diluted in PBS (1/1000, Invitrogen) for 1 h at room temperature and finally counterstained for 5 min with Hoechst 33258 (10 μg/ml in PBS, Sigma-Aldrich). Sections were then mounted onto Superfrost Plus glass slides in Fluoromount G mounting medium or Permount medium for Fluorojade-B protocol. For each section, images were taken using a 10× or 20× objective using the same microscope (Axioplan2, Zeiss) and camera (AxiocanMRm, Zeiss) settings. Fluorojade-B staining protocol has been performed following Schmued and Hopkins (2000).

### Quantification

KCC2 analysis was performed in blind. In control vehicle- and Y-27632-treated, pilocarpine vehicle- and Y-27632-treated rats, intensity and distribution analyses of KCC2 fluorescence, associated with the membrane or cytosolic regions, were performed on 50 CA3 pyramidal cells randomly selected for each animal condition (Table 2). On each cell, we applied the same straight line from the nucleus to the external cell compartment. The plot profile values were obtained using Plot analysis from Fiji software and the mean ± S.E.M are represented in the figure. The global fluorescence intensity analysis of KCC2 has been normalized to the number of cells contained in the ROIs. The 3D object counter pluggin from Fiji software was used to count the number of cells.

**Table 2:** **Details of animals, sections and ROIs used for the study**.

	Pilo + Veh	Pilo + ROCK-I	Ctrl + Veh	Ctrl + ROCK-I
Number of animals	16	24	12	16
Number of section per animal	5	5	5	5
Number of ROIs per animal (for KCC2 plot profile analysis)	50	50	50	50

### Statistical Analysis

Data are expressed as mean ± S.E.M. Statistical analyses were performed using GraphpadPrism (GraphPad software 6.01). For comparison between groups with normal distribution, the two-sample unpaired or paired Student’s *t* test was used for two groups. When data were not normally distributed, the Mann–Whitney rank-sum test (for unpaired data) was performed. For two-factor analysis, *p* values were determined by Tukey multiple-comparison test following two-way ANOVA analysis.

## Results

### Chronic Treatment with Y-27632 after SE Exacerbates Neuronal Cell Death During Epileptogenesis

**Figure 1:**
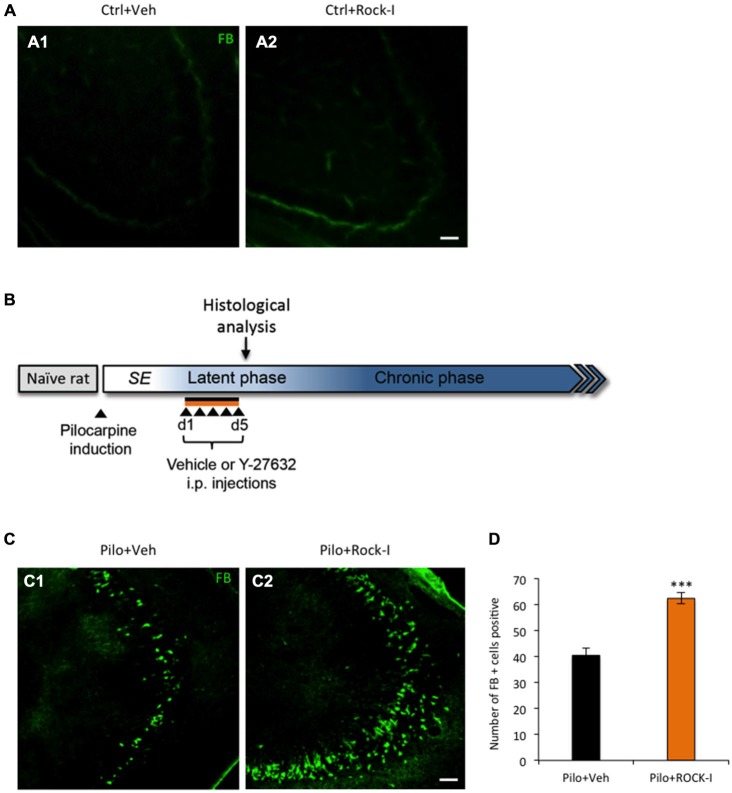
**Chronic treatment with Y-27632 after *Status Epilepticus* (SE) exacerbates neuronal cell death during epileptogenesis. (A)** Fluorojade B staining revealed no dead neurons in control vehicle- **(A1)** and Y-27632-treated animals **(A2)** (*n* = 3 for control vehicle-treated rats; *n* = 4 for control Y-27632-treated rats). **(B)** Experimental paradigm. Six weeks old male rats were subjected to 3 h of pilocarpine-induced SE. From day 1 to day 5 post SE, the rats were i.p. injected with either vehicle or Y-27632. **(C)** Fluorojade B positive cells (green) showing the dead neurons in pilocarpine vehicle- **(C1)** and Y-27632-treated animals **(C2)** in the CA3 region. **(D)** The histogram shows the quantification of the number of Fluorojade B positive cells (*n* = 3 for pilocarpine vehicle-treated; *n* = 4 for pilocarpine Y-27632-treated). Data are mean ± SEM; ***p < 0.001, *p* values obtained using *t* test analysis Scale bars = 20 μm.

The ROCK-signaling pathway is strongly activated in different trauma models including the kainate model of TLE and its acute inhibition has been shown to have a neuroprotective effect (Jeon et al., 2013; Rodriguez-Perez et al., 2013). However, from a clinical perspective it is relevant to investigate the influence of ROCK inhibition in the early post-insult time window. Thus, we examined the effect of daily i.p. administration of the selective ROCK inhibitor Y-27632 from day 1 to day 5 post-SE. In order to establish whether, under control condition, the chronic treatment of Y-27632 affects the cell neuronal death *in vivo*, we first analyzed the number of Fluorojade B-positive cells in the CA3 region of the hippocampus of control vehicle- and control Y-27632-treated rats (Figures 1A1,2). We observed no Fluorojade B-positive cells in the CA3 region of vehicle- and Y-27632-treated control rats. Similar results were found in the hilar and CA1 regions of the hippocampus (data not shown).

Next, we evaluate the effect of chronic Y-27632 treatment on cell death in a rat pilocarpine model (Figure 1B, experimental paradigm). In the Y-27632-treated pilocarpine rats we observed a statistically significant increase in the number of Fluorojade B-positive cells (Figure 1C2) as compared to pilocarpine vehicle-treated rats (Figure 1C1) at d5 post SE in the CA3 region of the hippocampus (Figure 1D; pilocarpine vehicle-treated rats 40.43 ± 2.71 vs. pilocarpine Y-27632-treated rats: 62.33 ± 2.12; ***p < 0.001). In contrary to the previously described acute neuroprotective effects of ROCK-inhibition, we found that the drug Y-27632 exacerbated neuronal cell death during early epileptogenesis in a rat pilocarpine model. Although the concentration used of the ROCK inhibitor is similar to previously reported, there is the possibility that the detrimental effect of Y-27632 are derived from concentration dependent nonspecific effects. For this reason we also assessed the effect of 5 mg/kg i.p. injection of ROCK inhibitor. We did not observed any effect on cell death in control treated or pilocarpine treated animal groups (pilocarpine vehicle-treated: 39 ± 2.43 vs. pilocarpine Y-27632-treated: 45 ± 2.32; *p* = 0.105, unpaired *t* test, data are mean ± SEM; data not shown).

### Y-27632 Treatment Increases Early Ectopic Recurrent Mossy Fiber Sprouting

**Figure 2:**
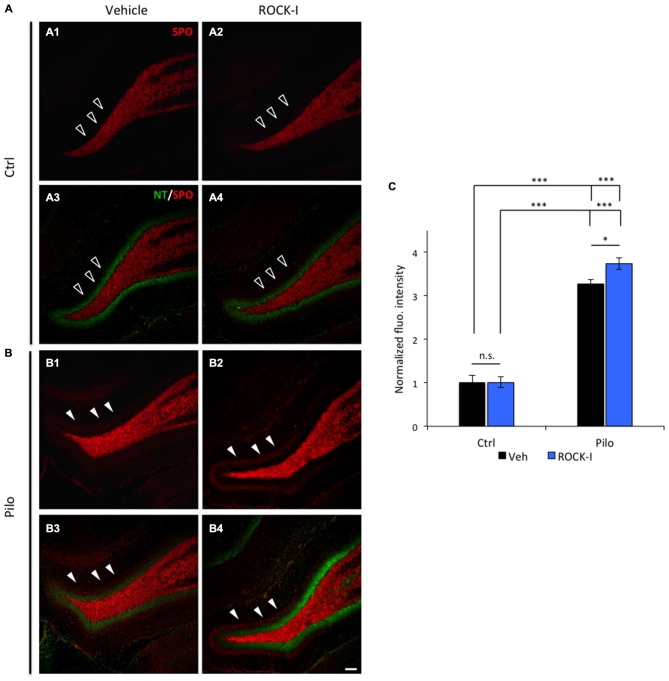
**Y-27632 treatment increases early ectopic recurrent mossy fiber sprouting (rMFS) in the dentate gyrus of pilocarpine rats.** Immunohistochemical staining with synaptoporin antibody (SPO, red) labeled the mossy fibers and neurotracer staining (NT, green) labeled cell body of the DG granule cells. **(A)** Synaptoporin staining revealed no ectopic rMFS band in the molecular layer of the DG in both ventral and dorsal DG in control vehicle- (**A1,3**; empty arrowheads) and Y-27632-treated rats (**A2,4**; empty arrowheads). **(B)** Synaptoporin staining revealed an ectopic rMFS band in the molecular layer of the DG in both ventral and dorsal DG in pilocarpine vehicle- (**B1,3**; arrowheads) and Y-27632-treated rats (**B2,4**; arrowheads). **(C)** The histogram represents the quantification of the fluorescence intensity of synaptoporin in control vehicle- and Y-27632-treated, and pilocarpine vehicle- and Y-27632-treated rats. The data are normalized to control vehicle-treated rats (*n* = 3 for control vehicle-treated rats; *n* = 4 for control Y-27632-treated rats; *n* = 3 for pilocarpine vehicle-treated; *n* = 4 for pilocarpine Y-27632-treated). Data are means ± SEM; *p < 0.05; ***p < 0.001, *p* values were determined by Tukey multiple-comparison test following two-way ANOVA analysis. Scale bar = 20 μm.

The ectopic recurrent MF sprouting (rMFS) within the dentate gyrus of the hippocampus is a major feature of TLE in both human patients and animal models (Tauck and Nadler, 1985; Represa et al., 1989; Sutula et al., 1989; Buckmaster et al., 2002; Buckmaster, 2012). This rMFS induces a strong recurrent excitatory circuit within the DG, and is partly responsible for the generation of epileptiform activities in human patients and animal models of TLE (Tauck and Nadler, 1985; Represa et al., 1989; Epsztein et al., 2005; Peret et al., 2014; Shima et al., 2015). Some studies suggest that rMFS is established during the early phase of epileptogenesis (Mathern et al., 1999; Harvey and Sloviter, 2005) and is a consequence of neuronal loss in the CA3 region (Shetty et al., 2005). Immunohistochemical visualization of synaptoporin (SPO) staining in the dorsal hippocampus (Volz et al., 2011; Peret et al., 2014) revealed a marked ectopic band within the molecular layer of DG in pilocarpine vehicle- and Y-27632-treated rats at d5pSE (as shown by the arrowheads in Figures 2B1,3 and Figures 2B2,4; the granular cell layer is labeled in green using the neurotracer marker, Figures 2A3,4–B3,4). As expected, rMFS was absent in control conditions (as shown by the empty arrowheads in Figures 2A1–4; both in control vehicle- and Y-27632-treated rats). The fluorescence intensity of the ectopic band within the molecular layer of DG was higher in pilocarpine Y-27632-treated rats as compared to pilocarpine vehicle-treated rats (Figure 2C; control vehicle-treated rats: 1.00 ± 0.17; control Y-27632-treated rats: 1.01 ± 0.09; pilocarpine vehicle-treated rats 3.27 ± 0.12; pilocarpine Y-27632-treated rats: 3.73 ± 0.13; *p < 0.05 and ***p < 0.001). Thus, these results are in accordance with an increased neuronal death in the CA3 region induced by ROCK inhibition in pilocarpine rats.

### Different Glial Response upon Y-27632 Treatment in Control and Post SE Conditions

The Rho-ROCK pathway seems to play a key role in neuronal survival and astroglial inflammatory responses (Kubo et al., 2008). Reactive gliosis and neuronal death are major events following SE and are thought to contribute to the pathogenesis of TLE (McNamara, 1994; Curia et al., 2008; do Nascimento et al., 2012). Despite the clear involvement of the Rho-ROCK pathway in microglia activation and astrogliosis, the qualitative effect of inhibiting this pathway may vary depending on the experimental model (for review, see Kubo et al., 2008; Fujita and Yamashita, 2014; Hensel et al., 2015). Moreover, few studies have demonstrated the implication of Rho-ROCK signaling pathway in neuronal survival and inflammatory response under epileptic conditions (İnan and Büyükafşar, 2008; Jeon et al., 2013). Thus, we investigated if the inhibitor Y-27632 influenced astrogliosis and if this could explain the increased neuronal cell death described at d5 post SE.

**Figure 3:**
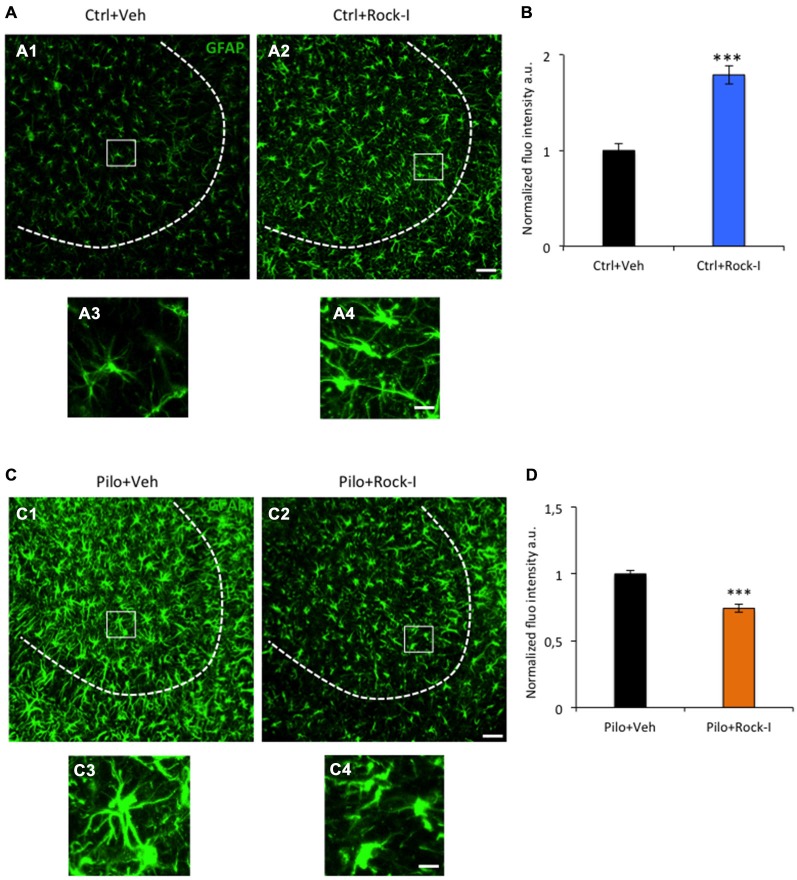
**Different glial responses upon Y-27632 treatment in control and post-SE conditions. (A)** Representative images of GFAP-immunopositive astrocytes (green) in control vehicle-treated **(A1,3)** and Y27632-treated **(A2,4)** rats. Dotted lines indicate the pyramidal cell layer of the CA3. The white squares in **(A1,2)** show higher magnifications of GFAP labeling **(A3,4)**. **(B)** The histogram represents the quantification of the fluorescence intensity (*n* = 3 for control vehicle-treated; *n* = 4 for control Y-27632-treated). Data are means ± SEM; ***p < 0.001, *p* values obtained using Mann–Whitney rank-sum test analysis. **(C)** Representative images of GFAP-immunopositive astrocytes (green) in the CA3 region in pilocarpine vehicle-treated **(C1,3)** and pilocarpine Y27632-treated **(C2,4)** rats. Dotted lines indicate the pyramidal cell layer of the CA3 region. The white squares in **(C1,2)** show higher magnifications of GFAP labeling **(C3,4)**. **(D)** The histogram represents the quantification of the fluorescence intensity (*n* = 4 for pilocarpine vehicle-treated; *n* = 8 for pilocarpine Y-27632-treated). Data are means ± SEM; ***p < 0.001, *p* values obtained using Mann–Whitney rank-sum test analysis. Scale bars: A1, 2; C1, 2 = 20μm. A3, 4; C3, 4 = 15 μm.

We first studied the effect of Y-27632 treatment on astrogliosis on control rats. Surprisingly, we observed a significant increase in GFAP fluorescence intensity in Y-27632-treated control rats (Figure 3A2) as compared to vehicle-treated control rats (Figure 3A1; Figure 3B, control vehicle-treated rats 1.00 ± 0.07 vs. control Y-27632-treated rats: 1.78 ± 0.09; ***p < 0.001). The chronic ROCK inhibitor treatment also altered the morphology of GFAP-positive astrocytes (Figures 3A3,4). The astrocytes had a more elongated shape, with long processes (Figure 3A4). In conclusion, our results show that the chronic ROCK inhibitor Y-27632 treatment under control conditions increased the astrocyte-mediated inflammatory response which was interestingly not concomitant to neuronal death.

Next, we evaluate the effect of chronic Y-27632 treatment on astrogliosis in the rat pilocarpine model (see Figure 1B, experimental paradigm). Unlike in control conditions, we showed that reactive astrogliosis was substantially decreased in pilocarpine Y-27632-treated rats at d5 post SE rats (Figure 3C2) as compared to pilocarpine vehicle-treated rats (Figure 3C1) (Figure 3D; pilocarpine vehicle-treated rats 1.00 ± 0.02 vs. pilocarpine Y-27632-treated rats: 0.74 ± 0.02; ***p < 0.001). This was accompanied by morphological changes of GFAP-positive astrocytes after ROCK inhibition, with a retraction of the processes and astrocytes having an amyboïd shape (Figures 3C3,4). Altogether, we showed that chronic ROCK inhibitor Y-27632 treatment reduces astrocyte density during epileptogenesis in the pilocarpine rat model. Thus, these data disclose an intricate relationship between changes in astroglial morphology and SE-induced cell death and indicate that the effect of ROCK inhibition on neuronal cell death may largely not depend on astroglial changes.

### Y-27632 Treatment During Epileptogenesis Alters KCC2 Expression

**Figure 4:**
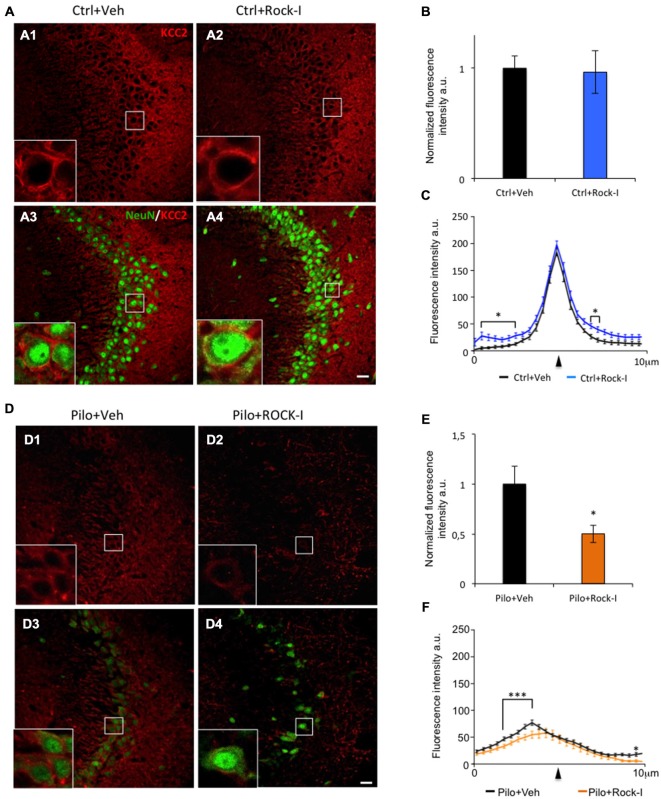
**Y-27632 treatment during epileptogenesis alters KCC2 expression in the CA3 region. (A)** Immunohistochemical stainings with KCC2 antibody (red) and NeuN (green) in the CA3 region of control vehicle- **(A1,3)** and Y-27632-treated animals **(A2,4)**. The white squares in **(A1–4)** show higher magnifications of KCC2 labeling. **(B)** The histogram represents the quantification of the fluorescence of KCC2 labeling in control vehicle- and Y-27632-treated animals. The data are normalized to control. **(C)** The curves represent the plot profile of KCC2 staining along 10 μm from the nucleus to the external neuropil of the neurons analyzed. The arrowhead indicates the putative membrane location (*n* = 3 for control vehicle-treated; *n* = 4 for control Y-27632-treated). **(D)** Immunohistochemical stainings with KCC2 antibody (red) and NeuN (green) in the CA3 region in pilocarpine vehicle- **(D1,3)** or with Y-27632-treated animals **(D2,4)**. The white squares in **(D1–4)** show higher magnifications of KCC2 labeling. **(E)** The histogram represents the quantification of the fluorescence of KCC2 labeling in pilocarpine vehicle- or with Y-27632-treated animals. The data are normalized to pilocarpine vehicle-treated rats. **(F)** The curves represent the plot profile of KCC2 staining along 10 μm from the nucleus to external neuropil of the neurons analyzed (*n* = 4 for pilocarpine vehicle-treated; *n* = 8 for pilocarpine Y-27632-treated). Data are means ± SEM; *p < 0.05; ***p < 0.001, *p* values obtained using *t* test analysis. Scale bars = 20 μm.

In addition to the neuronal loss and reactive gliosis, TLE is also characterized by an impaired GABAergic inhibition. KCC2 is one of the chloride co-transporter responsible for establishing the neuronal Cl^−^ gradient that governs GABAergic inhibition. Under epileptic conditions, KCC2 is downregulated which leads to impaired GABAergic inhibition in both human patients and animal models of TLE (Palma et al., 2006; Huberfeld et al., 2007; Pathak et al., 2007; Bragin et al., 2009; Barmashenko et al., 2011). Importantly, recent results directly involve KCC2 in the post-traumatic events leading to neuronal cell lost (Pellegrino et al., 2011; Winkelmann et al., 2015). With this as a background, we assessed the effect of ROCK inhibitor Y-27632 on KCC2 expression under control and epileptic conditions. In the CA3 region of control vehicle- and Y-27632-treated rats, KCC2 immunostaining was strong (Figures 4A1,2); the intensity of staining was not significantly different between the two groups (Figure 4B; control vehicle-treated rats 1.00 ± 0.07 vs. control Y-27632-treated rats: 0.86 ± 0.02; *p* > 0.05). In controlY-27632-treated rats, similar intensity and pattern of expression was observed (Figures 4A3,4). At higher magnification, we can see that KCC2 immunolabeling is expressed in the perisomatic region of CA3 pyramidal cells (stained using NeuN antibody) in both conditions (Figures 4A1,2 and insert boxes). To confirm these observations, we analyzed the intensity and repartition of KCC2 plotted as a function of pixel distance across the soma. The overall pattern of expression of KCC2 is comparable in control vehicle- and Y-27632-treated rats with a narrow peak at the perisomatic level (Figure 4C, arrowhead); with a significantly but minor difference in the cytoplasm and in the external part of the cells (Figure 4C, asterisks, *p* < 0.05). Those results prompt us to conclude that ROCK inhibitor Y-27632 has no overall effect on KCC2 expression pattern under control conditions.

We next investigated the effect of ROCK inhibitor Y-27632 treatment on KCC2 expression in the pilocarpine rats. Interestingly, we observed a lower KCC2 immunoreactvity in the CA3 pyramidal layer as well as in layers containing the dendritic trees (stratum oriens and stratum radiatum) in pilocarpine Y-27632-treated rats (Figures 4D2,4) as compared to pilocarpine vehicle-treated rats (Figures 4D1,3). These observations were confirmed by the analysis of fluorescence intensity showing a significantly decreased intensity in pilocarpine Y-27632-treated rats as compared to pilocarpine vehicle-treated rats (Figure 4E; pilocarpine vehicle-treated rats 1.00 ± 0.08 vs. control Y-27632-treated rats: 0.53 ± 0.04; *p < 0.05). The intensity and repartition analysis of KCC2 staining show that KCC2 was very low and diffuse within the neuron cell bodies in both conditions (Figures 4D,F). In pilocarpine Y-27632-treated rats, the cytoplasmic levels of KCC2 immunostaining were significantly decreased as compared to pilocarpine vehicle-treated (Figure 4F, ***p < 0.01). Moreover, we also assessed the effect of 5 mg/kg i.p. injection of ROCK inhibitor and did not observed any effect on KCC2 expression in control treated or pilocarpine treated animal groups (control vehicle-treated: 1.00 ± 0.09; control Y-27632-treated: 1.03 ± 0.12; *p* = 0.65, unpaired *t* test; pilocarpine vehicle-treated: 1.00 ± 0.11 vs. pilocarpine Y-27632-treated: 0.90 ± 0.11; *p* = 0.54, unpaired *t* test, data are mean ± SEM; data not shown).

Altogether, these results show that chronic ROCK inhibitor Y-27632 treatment has a detrimental effect on KCC2 expression during epileptogenesis and not under control conditions. Thus, we found that chronic ROCK inhibition treatment exacerbates the pilocarpine-induced down-regulation of KCC2 and this could play an important role in the mechanism mediating the neuronal loss induced by this inhibitor during epileptogenesis.

## Discussion

The RhoA pathway and its downstream effector ROCK are activated under various pathological conditions including Alzheimer’s (Zhou et al., 2003) and Parkinson’s diseases (Rodriguez-Perez et al., 2013), spinal cord injury (Fournier et al., 2003), global cerebral ischemia (Castro-Alvarez et al., 2011), traumatic brain injury (Dubreuil et al., 2006) as well as in epilepsy (Dubreuil et al., 2006; İnan and Büyükafşar, 2008; Jeon et al., 2013). Besides, the ROCK signaling pathway is involved in numerous fundamental cellular functions such as cell adhesion, apoptosis, inflammatory responses and neurite outgrowth (see for review, Kubo et al., 2008; Fujita and Yamashita, 2014; Hensel et al., 2015). To this end, several drugs such as Fasudil or Y-27632 have been developed to inhibit ROCK activity in order to treat the diverse pathological conditions where RhoA-ROCK signaling pathway is activated (Kubo et al., 2008). Importantly, ROCK inhibition using Y-27632 has an acute antiepileptic effect in the PTZ model of epilepsy (İnan and Büyükafşar, 2008), as well as a neuroprotective effect in KA-induced SE when applied acutely prior to the SE (Jeon et al., 2013). Thus, although the acute effect of ROCK inhibition is established, it was desirable to evaluate the impact of the use of inhibitors of this pathway in the early post-SE time window.

In the present study, we have evaluated the effect of chronic ROCK inhibitor Y-27632 treatment during the early phase of epileptogenesis in the rat pilocarpine model. Interestingly, our results uncovered a differential effect of ROCK inhibitor treatment between control and epileptic conditions. Unlike the positive acute effect of ROCK inhibition when applied prior to the onset of SE, we found that chronic Y-27632 treatment after SE resulted in a significant increase in cell death in pilocarpine rats which was followed by augmented ectopic rMFS during early epileptogenesis at d5 post SE. These results could no be explained by an increased reactive astrogliosis after ROCK inhibition as the specific compound Y-27632 reduced reactive astrogliosis during epileptogenesis. Interestingly, we found that Y-27632 treatment exacerbates KCC2 downregulation, a protein that has been recently linked with neuronal cell death (Pellegrino et al., 2011; Winkelmann et al., 2015). However, in control rats, this chronic treatment did not affect cell survival, KCC2 expression, or the rMFS, but increased astrocyte density.

One of the hallmarks of TLE is the ectopic rMFS described in the molecular layer of the dentate gyrus within the hippocampus (Tauck and Nadler, 1985; Represa et al., 1989; Gabriel et al., 2004; de Lanerolle et al., 2012; Peret et al., 2014; Shima et al., 2015). ROCK activity is involved in the axonal outgrowth and its inhibition, using Y-27632, induces axonal regeneration after spinal cord injury (Fournier et al., 2003). This prompts us to evaluate the extent of ectopic rMFS after chronic Y-27632 treatment during epileptogenesis. Here we have shown that Y-27632 treatment increased ectopic rMFS during early epileptogenesis. The cellular and molecular mechanisms underlying this effect remain unclear. The extent of rMFS has been correlated to seizure frequency (Sutula et al., 1989), mossy cell loss (Houser, 1990; Nadler, 2003; although recently challenged by Jinde et al., 2012) and more importantly to CA3 cell loss (Shetty et al., 2005). Thus, in the present study, the effect of ROCK inhibition on ectopic rMFS during epileptogenesis could also partly reflect the increased cell loss in the CA3 in the pilocarpine model.

Although ROCK inhibition is an attractive therapeutic target, the cellular and molecular mechanisms requiring this pathway are far from being fully understood. ROCK activity is known to be involved in neuronal cell death as well as astroglial and microglial neuroinflammatory responses (Kubo et al., 2008). The present findings show that ROCK inhibition using Y-27632 reduced the reactive astrogliosis at d5 post SE in the pilocarpine rats. However, Y-27632 treatment under control conditions increased the density and modified the morphology of astrocytes. A closer look at previous work shows, in fact, that both effect have been observed depending on the pathology and model considered. For instance, in hypoxia-reoxygenation injury (Ding et al., 2010) and in neurodegenerative diseases such as Parkinson’s disease (Barcia et al., 2012), ROCK inhibition resulted in a reduction of microglia while in a SOD mouse model of amyotrophic lateral sclerosis (ALS) the ROCK inhibitor Fasudil induced an increase in microglia density and a reduction of reactive astrogliosis (Tönges et al., 2014). Conversely, in a spinal cord injury model, the inhibition of ROCK using Y-27632 resulted in an increase of astrogliosis (Chan et al., 2007). Also the results obtained in the present study in sham treated animals are supported by previous studies reporting that, under control conditions, either Fasudil or Y-27632 increased astrocyte density *in vivo* (Chan et al., 2007); *in vitro*, they induced an increase of viability and morphological changes of astrocytes (Lau et al., 2011; O’Shea et al., 2015). Thus, the opposite effects of Y-27632 obtained under control and post-SE conditions suggest that the same pathway is recruited for different cellular processes in a context dependent manner.

Reactive gliosis has been proposed to have positive and negative effects after CNS injury (Sofroniew, 2005; Losi et al., 2012; Burda and Sofroniew, 2014; Jones, 2015). The astrogliosis could mediate pro- as well as anti-inflammatory responses and, in turn, have the potential for neural toxicity as well as neuroprotection. Hence, the increased cell death could be a consequence of a reduced density of reactive gliosis that is known to help for the clearance of extracellular space, especially during insults (Bush et al., 1999). Thus, the relationship between plastic changes in astroglia and ROCK inhibition inducing an increase of cell death appears to be highly complex.

In our search for a mechanism for the increased neuronal death following Y-27632 treatment, we ask if post-SE changes in GABAergic transmission could be involved. An important factor contributing to epileptogenesis is the excessive intracellular accumulation of chloride. This leads to compromised GABAergic inhibition and hyperexcitability in TLE (Palma et al., 2006; Huberfeld et al., 2007; Muñoz et al., 2007; Bragin et al., 2009; Barmashenko et al., 2011). KCC2 is a major neuronal chloride co-transporter which is responsible for establishing the neuronal Cl^−^ gradient that governs GABAergic inhibition (Medina et al., 2014). Some studies have shown that this transporter is downregulated during epileptogenesis in TLE models of epilepsy (Pathak et al., 2007; Bragin et al., 2009; Barmashenko et al., 2011). In our recent publications we have demonstrated that: (i) GABA_A_-R mediated depolarization triggers RhoA-ROCK activation in an *in vitro* axotomy model (Shulga et al., 2012) and (ii) an interaction between KCC2 and small Rho GTPases influence actin dynamics and spinogenesis (Llano et al., 2015). In the present study, we show that the treatment with Y-27632 for a few days post SE had no effect under control condition but could delay the post SE KCC2 recovery or exacerbate KCC2 downregulation in pilocarpine rats. Previous reports have shown that excessive glutamate levels can lead to loss of KCC2 expression and function and the abolition of the hyperpolarizing effect of GABAergic transmission (Lee et al., 2011). Importantly, recent results directly linked KCC2 to proapoptotic mechanisms. Pellegrino et al. (2011) have shown that KCC2 loss increases cell vulnerability to toxicity and subsequent cell death. In this study, the authors demonstrated a correlation between the survival rate of neurons after excitotoxic insult *in vitro* (30 min of 40 μM NMDA application in cultured hippocampal neurons) and the recovery of KCC2 expression within the 24 h following the insult (Pellegrino et al., 2011). Moreover, Winkelmann and collaborators showed an important role of the N-terminal domain of KCC2 in glutamate induced neuronal apoptosis (Winkelmann et al., 2015). Thus, our data on KCC2 expression in pilocarpine rats suggest that the ROCK signaling pathway could be involved in the recovery of KCC2 after an excitotoxic insult, such as an SE episode, and that this delay subsequently induce neuronal loss. This is interesting in view that studies have shown a recovery of KCC2 expression that could be cell type specific (e.g., Huberfeld et al., 2007). Thus, it will be important to investigate whether differential ROCK signaling could be involved in this phenomenon.

The underlying mechanisms of KCC2 downregulation seem to involve the phosphorylation state of KCC2 (Lee et al., 2011; for review, see Medina et al., 2014). Interestingly, in a recent report, the activation of RhoA-ROCK-PTEN pathway was shown to decrease the phosphorylation levels of GABA_A_-Rs and to trigger endocytosis and degradation of internalized receptors (Riffault et al., 2014). A similar mechanism could affect the expression of KCC2 in the presence of the inhibitor Y-27632.

In conclusion, our findings evaluate for the first time the chronic use of the ROCK inhibitor Y-27632 as a post SE prophylactic drug during epileptogenesis in the pilocarpine model. Our study suggests that this treatment has an overall detrimental effect on major morphological features and neuronal survival after SE. While the data show an intricate involvement of astrogliosis, they suggest that post SE activation of the ROCK pathway acts to maintain KCC2 expression. This may, in turn, constitute part of a pro-survival mechanism during the epileptogenic window following SE. Further investigations are necessary to decipher, in more detail, the mechanisms of ROCK activation in astrogliosis, rMFS and KCC2 expression in the events leading to the development of chronic epilepsy.

## Conflict of Interest Statement

The authors declare that the research was conducted in the absence of any commercial or financial relationships that could be construed as a potential conflict of interest.
